# Upregulated expression of serum exosomal miR-375 and miR-1307 enhance the diagnostic power of CA125 for ovarian cancer

**DOI:** 10.1186/s13048-018-0477-x

**Published:** 2019-01-22

**Authors:** Ying Ying Su, Li Sun, Zhi Rui Guo, Jin Chang Li, Ting Ting Bai, Xiao Xiao Cai, Wen Han Li, Ye Fei Zhu

**Affiliations:** 1 The Second Affiliated Hospital, Nanjing Medical University, Nanjing, 210011 Jiangsu China; 2 The Affiliated Hospital of Nanjing University of Chinese Medicine, Jiangsu Province Hospital of Traditional Chinese Medicine, Nanjing, China

**Keywords:** Ovarian cancer, Serum exosome, microRNA, Biomarker

## Abstract

**Background:**

Ovarian cancer (OC) is associated with high mortality in gynecological oncology; this is mainly due to the low diagnosis rate. Exosomal miRNA has demonstrated potential as a tumor biomarker. We aimed to explore the diagnostic potential of serum exosomal miR-1307 and miR-375 for OC.

**Methods:**

The first six candidate miRNAs were selected from the previous literature. The relative quantification of qRT-PCR was used to screen for the stability of exosomal miRNAs, followed by validation of the cohort. ROC analysis was employed to analyze the specificity and sensitivity of exosomal miRNA.

**Results:**

MiR-1307 and miR-375 were confirmed stably existing in serum exosomes of OC. Moreover, miR-1307 and miR-375 were both significantly up-regulated in serum exosomes of OC compared to ovarian benign and healthy groups. The overexpressed miRNAs showed independent diagnostic power and enhanced the diagnostic accuracy of traditional biomarkers when combined with CA-125 and HE4. MiR-1307 was associated with tumor staging, and miR-375 was associated with lymph node metastasis of OC.

**Conclusion:**

Our results suggest that serum exosomal miR-1307 and miR-375 could serve as potential tumor biomarkers to improve diagnostic efficiency for OC.

## Introduction

Ovarian cancer is a serious gynecologic disease. The incidence of OC is the second highest among gynecological tumors, accounting for 3% of all tumors. However, the mortality rate is approximately 5%, meaning that about 225,500 new cases are reported annually, resulting in over 140,200 deaths worldwide [1–3]. Diagnosis in early stage is not common for OC, because of the lack of specific signs and symptoms in early stage [4]; However, abdominal mass and ascites, abdominal pain often suggest advanced development of OC. The conventional serum tumor biomarker CA125 combined with abdominal and pelvic, transvaginal ultrasonography were usually performed for the initial diagnostic of ovarian cancer in clinical [5]. However, serum CA125 level abnormal in not just 80% of OC but menopause and benign gynecological disease [6]. HE4 is restrictive in the diagnosis of OC due to its sensitivity to multiple abdominal tumors such as Primary peritoneal carcinoma, primary fallopian tube cancer [7, 8]. The absence of specific symptoms during the early stages of the disease combined with the lack of efficient biomarkers results in 70% of patients being diagnosed only in the later stages [9]. The primary therapy is laparoscopic surgery in the early stage [10] and radical cell resection followed by postoperative chemotherapy in late stage [11]. Prognosis in OC depends largely on the stage at the time the disease is confirmed. The commonly observed chemoresistance in stages III and IV is associated with a five-year survival rate of less than 30%; however, patients with stage I and II have a better prognosis of more than 90% five-year survival [12]. New diagnostic strategies continue needed to be explored to meet the challenge of OC.

Exosomes were discovered in 1983, but first named as such in 1987 by Johnstone et al. [13] . Exosomes belong to a large family of membrane vesicles, the term specifically refers to nanosized membrane-enclosed vesicles ranging from 30 to 150 nm [14] and having a classic “cup” or “dish” morphology [15]. Derived from varies types of cells, exosomes exist in various kinds of body fluids such as serum, urine, saliva, ascites fluid, pleural fluid, and breast milk [16]. Exosomes contain a variety of biomolecules, including lipids, proteins, DNAs, and RNAs [17, 18], and the cargoes within exosomes were thought to be selectively packaged according to the originating cell and to function in types of cell communication that are related to disease characteristics [19, 20]. Recent studies have demonstrated that exosomes are involved in intercellular communication and mediation in both physiological and pathological processes [21]. Specific miRNA such as miR-151a, miR-9, let-7 and miR-200, which play role in drug-resistance, tumor invasiveness, angiogenesis have been reported in exosomes [22–24]. Moreover, differentially expressed serum exosomal miRNA could act as tumor biomarker, such as miR-223, miR-425-3p [25, 26]. Exosomes are now frequently used in liquid biopsies, and they have profound potential in targeted treatment.

MicroRNAs (miRNAs) refer to a class of noncoding single-stranded RNA molecules 19 to 22 nucleotides in length [27]. MiRNAs play significant roles in inhibiting mRNA translation and mRNA degradation, and they are involved in regulating many cellular processes both physiologically and pathologically [28]. MiRNA expression profiling of tissue and serum have been widely carried out. Dysregulated miRNAs have been proved to be predictor of disease progression, prognosis and metastasis and may serve as molecular biomarkers for disease detection [29–31]. Tissue miRNAs are invasive, and both tissue- and serum-derived miRNAs present poor stability in the presence of interference factors. However, exosomal miRNAs in serum are thought to be more stable, and thus could be used in medical applications [32]. Here, we explored the application significance of exosomal miRNAs as biomarkers for the diagnosis of OC.

## Materials and methods

### Patients and samples

The study population was composed of 50 OC patients, 50 healthy volunteers, and 50 benign ovarian tumor patients. The OCand benign ovarian tumor assignments were confirmed by pathological diagnosis of surgical specimens. The age of study participants ranged from 21 to 83 years, and the median age was 49 years. Clinical characteristics of the cancer patients enrolled in this study are listed in Table 2.

### Serum

All of the serum samples were obtained prior to surgical operations in the second affiliated hospital of the Nanjing Medical University. Serum was separated by centrifugation and then stored at  -80 °C.

### Exosome isolation

Exosomes were isolated from 0.5 ml serum using the Total Exosome Isolation Reagent (Invitrogen, USA) according to the manufacturer’s protocol. The serum was thawed on ice at 25 °C and then subjected to centrifugation at 2000 g for 30 min to remove possible residual cell debris. Next, 500 μL of serum supernatant was mixed with 100 μL reagent, followed by incubation for 30 min and centrifugation at 10,000 g for 10 min. The supernatant was removed, and total exosomes dissolved in 100 μL PBS in preparation for the next RNA extraction.

### Transmission electron microscopy

Exosome pellets were resuspended in PBS, and the solution was dropped onto a carbon-coated copper grid with a mesh diameter of 2 nm for 2 min. The excess liquid was removed, and filter paper was used to drain the grid; a drop was negatively stained with phosphotungstic acid and loaded onto the grid for 5 min. The grid was then dried at room temperature. Finally, the samples were observed under a JEOL JEM-1010 transmission electron microscope (Japan JEOL; 2000) operated at 80 kV.

### Nanoparticle-tracking analysis (NTA)

After being collected by the Total Exosome Isolation Reagent as above and diluted 2000 times in particle-free PBS, the exosomes were injected into the NanoSight sample pool. Next, the exosome size distribution and concentration were analyzed using a NanoSight NS300 Instrument (Malvern Instruments, Malvern, UK) equipped with NTA 2.3 software. Sixty-second videos were recorded, and exosomes were sized and counted using the NTA 2.3 software.

### Western blotting

Exosomes were processed with RIPA lysis buffer to obtain the exosomal proteins, and the proteins were quantified via the BCA method. According to gradient from 15 μg to 30 μg exosomal protein was loaded onto sodium dodecyl sulfate-polyacrylamide gels for electrophoresis (SDS-PAGE), then transferred to a PVDF membrane. The PVDF membrane was blocked with BSA at room temperature for one hour and incubated with primary antibodies (CD-63) overnight. The membranes were then incubated with the corresponding HRP-conjugated secondary antibody. The transferred protein on the PVDF membrane was finally detected by chemiluminescence using the BIO-RAD Chemidoc XRS system.

### MicroRNA isolation and real-time RT-PCR assay

Total RNA was extracted from serum exosomes using the TRIzol Reagent (Invitrogen, USA) following the manufacturer’s instructions. The isolated RNA was diluted in 14 ul DNase and RNase-free water. RNA quantity and quality were detected using an OD-1000+ Spectrophotometer (one drop, USA). Ninety nanograms of total RNA were converted into cDNA, one microliter cDNA product were used for the qRT-PCR in the final volume of 10 μl. MiRNA-39 was used as an internal control. Total RNA was reverse transcribed into cDNA using the HiScript® II Q RT SuperMix for qPCR (Vazyme Biotech Co. Ltd., China) performed on the S1000 Thermal Cycler (BIO-RAD, USA). The measurement of miRNA expression level was conducted by relative quantitative real-time PCR. The qRT-PCR was carried out using SYBR Green master mix (Vazyme Biotech Co. Ltd, China) on the StepOnePlus™ (ABI, USA). The designed primers of miRNAs are listed in Table 1. The reactions were incubated in a 96-well plate at 95 °C for 10 min, followed by 40 cycles at 95 °C for 10 s, annealing at 60 °C for 20 s, and elongation at 72 °C for 10 s. MiRNA relative expression level was calculated using the 2 ^- ΔΔ Ct^ method.

**Table 1:** Primers used for RT-qPCR

Primer	Forward Sequence (mature miRNA)	Reverse Sequence
miR-1307	5’-AACTCGGCGTGGC -3’	5’-GAGCAGGCTGGAGAA-3’
miR-375	5’-AGTTTGTTCGTTCGGCTC-3’	5’-GTGCAGGGTCCGAGGT -3’
miR-146	5’ -GGGTGAGAACTGAATTCCA-3’	5’ -CAGTGCGTGTCGTGGAGT-3’
miR-214	5’ -ACAGCAGGCACAGACAGGCAGU-3’	5’ -UGCCUGUCUGUGCCUGCUGUUU -3’
miR-130a	5’ -ACACTCCAGCTGGGTTCACATTGTGCTACTGT-3’	5’ -TGTCGTGGAGTCGGCAATTC -3’
miR-130b	5’-GGGCAGTGCAATGATG-3’	5’-GTGCGTGTCGTGGAGTCG-3’

### Statistical analysis

The statistical analysis was performed using the SPSS 23.0 statistical software. The differences in serum exosomal miRNA levels were compared by Mann–Whitney U tests. Relative exosomal miRNA expression was calculated using the ΔΔCT method (ΔCT = CT miR − CT reference). All of the experimental data are shown as the mean ± SE. The area under the curve (AUC) was applied to access diagnostic power of exosomal miRNAs. A *P* value of less than 0.05 was considered to be significant in all statistical analyses.

## Results

### Evaluation of circulating serum exosomes

**Fig. 1:**
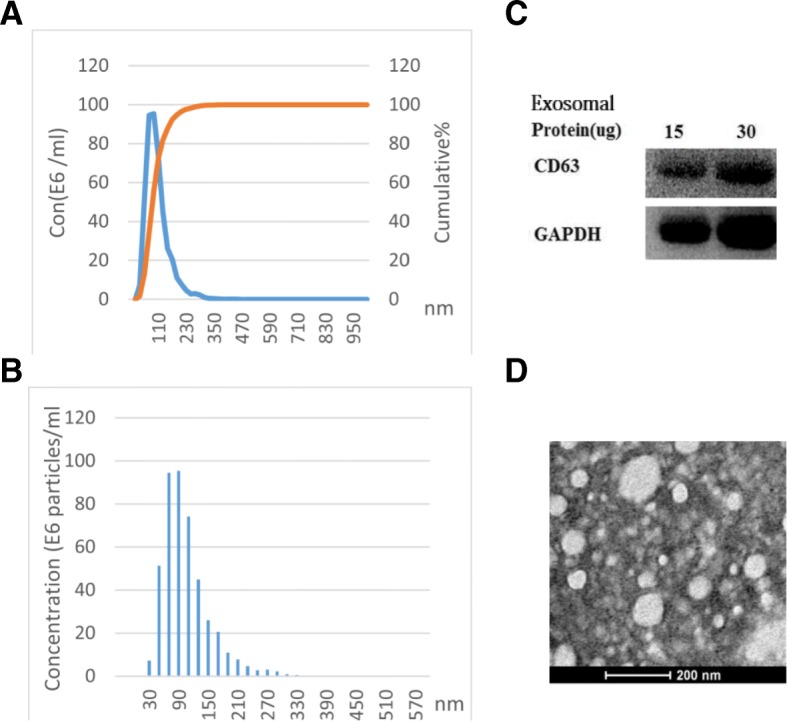
Serum exosome characteristics. **a**, **b** The Nanoparticle-tracking analysis (NTA) presents the size and concentration distribution. Serum exosomes were diluted 2000 times after extraction according to the protocol. **c** Western blotting showed the presence of CD63 in the exosome membrane. GAPDH was used as a control. **d** Transmission electron microscopy (TEM) revealed the round shape of exosomes by negatively staining the background with phosphotungstic acid. The bar represents 200 nm

The following analysis was used to confirm the quality and efficiency of the isolated serum exosomes. The nanoparticle-tracking analysis (NTA) showed that the diameter peaked at 110 nm, with a mean of 105 nm (Fig. 1a). The size distribution of exosomes ranged from 30 to 170 nm (Fig. 1b). Transmission electron microscopy (TEM) analysis revealed the round shape of exosomes that were derived from serum (Fig. 1d) and that the particle diameter observed in the photographs was in accord with the NTA. Furthermore, we detected the exosome membrane-specific protein marker CD63 by Western blot analysis (Fig. 1c). Combined with the results of the various methods confirmed the successful extraction of serum exosomes.

### MiR-1307 and Mir-375 can distinguish OC from benign tumors and healthy controls

**Fig. 2:**
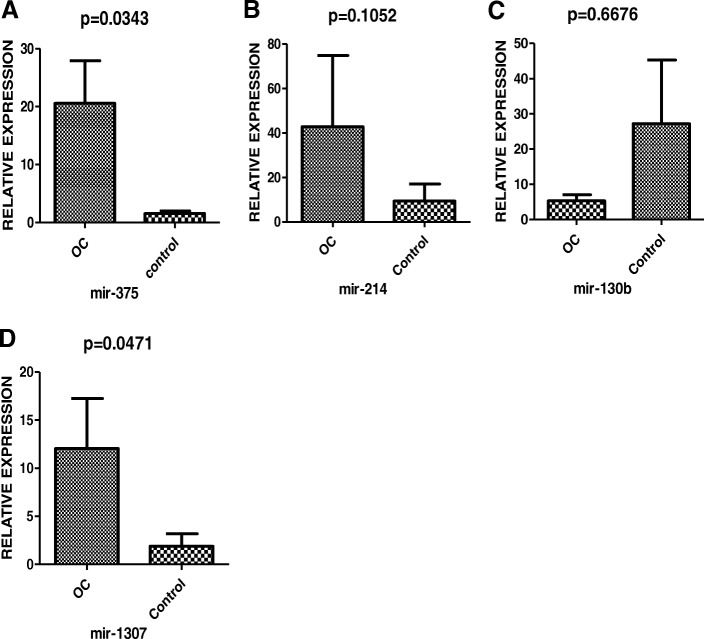
The relative expression levels of six selected miRNAs between OC and healthy control were analyzed by the qRT-PCR. The miR-1307 (D), miR-375 (*p* = 0.0471, *p* = 0.0343, *P* < 0.05) (A) were screened for the further cohort validation

**Fig. 3:**
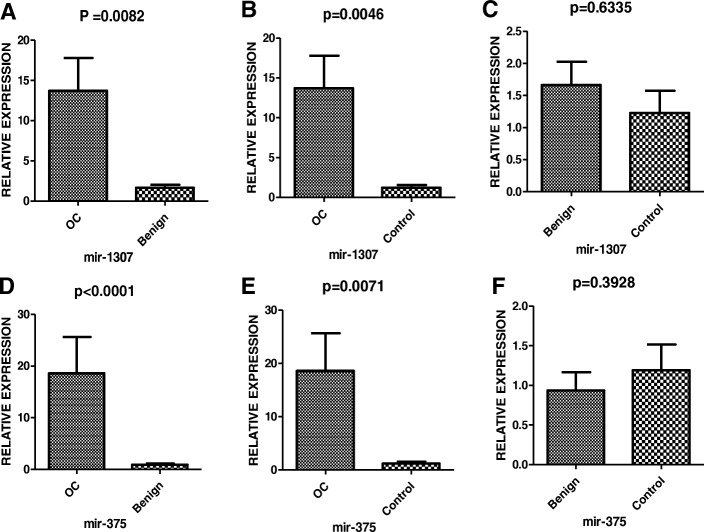
Relative serum exosome expression level of candidate miR-1307 (A, B, C) and miR-375 (D, E, F). The cel-miR-39 was used as an internal control. The *P* values were determined by the Mann–Whitney U test. OC = ovarian cancer

In the following study, six miRNA candidates (miR-1307, miR-130a, miR-130b, miR-214, miR-375, and miR-146) were selected for validation among the patients suffering from OC. The patients’ clinical characteristics and information obtained are listed in Table 2. Healthy individuals with ages identical to OC patients and benign ovarian tumor cases were used as controls. The real-time relative quantification to investigate exosomal miRNA stability was performed only for the OC and healthy control groups. Mir-130a could barely be detected, while miR-146 showed unstable amplification. MiR-130b miR-214, miR-1307, and miR-375 could be stably amplified in serum exosomes. MiR-130a (Fig. 2c), and miR-214 (Fig. 2b) showed no significant difference; however, miR-1307 (Fig. 2d) and miR-375 (Fig. 2a) were up-regulated in the OC group. Further analysis of miR-1307 and miR-375 was conducted in the OC , benign ovarian tumor, and healthy control groups. After being normalized by cell-miR-39, the identification of the relationship between OC and miRNA markers uncovered a statistically significant increase in both miR-1307 and miR-375 in OC patients when compared with healthy control and benign ovarian disease (all *P* < 0.05; Fig. 3a, b, d, e). However, no significant differences were discovered between benign ovarian disease and healthy controls (all *P* > 0.05; Fig. 3c, f).

**Table 2:** Clinicopathological features of the cohort study

Clinicopathological features		Number of patients
	Range	21-85
Age	≥50,n (%)	68; 59.13%
	<50, n(%)	47; 40.87%
Histology	Serous	50
	Benign	40
	Control	25
OC FIGO stage	I	4
	II	4
	III	20
	IV	22
Lymph node metastasis	Negative	15
	Positive	35
Residual tumor	Yes	50
	no	0
Position, n (%)	Bilateral	32; (65.7%)
	Unilateral	18; (34.3%)

### MiR-1307 strengthens the diagnostic capacity of CA-125 and HE4 for OC 

**Fig. 4:**
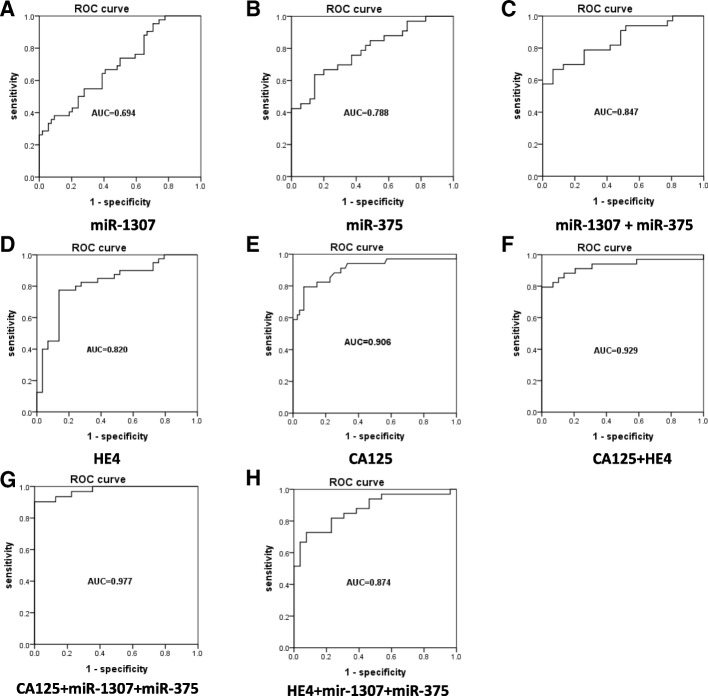
The diagnostic value of different biomarkers from the ROC curve. (D, E) and (A, B) show the diagnostic power of serum biomarkers and serum exosomal miRNA, respectively. (C, F) The exosomal miRNA enhanced the diagnostic power of the candidate biomarker panel. (G, H) Significant increase of efficiency of diagnosis when combining the new exosomal miRNAs biomarkers with CA125 and HE4

To evaluate the diagnostic power of serum exosome miRNA and conventional serum tumor biomarkers CA125 and HE4, an ROC curve analysis was performed (see Fig. 4 and Table 3). CA125 had an area under the ROC curve (AUC) value of 0.906 (Fig. 4e, *P* < 0.0001), and for HE4 the AUC = 0.820 (Fig. 4d, *P* < 0.0001). The AUC value for miR-1307 = 0.694 (Fig. 4a, *P* = 0.0054), and for miR-375 the AUC = 0.788 (Fig. 4b, *P* < 0.0001). The combination of the conventional serum tumor biomarkers of CA125 and HE4 generated an increased diagnostic power AUC of 0.929 (Fig. 4f, *P* < 0.0001). Significant synergistic effects were observed in the upregulation of miR-1307 combined with miR-375, which generated an AUC of 0.837 (Fig. 4c, *P* < 0.0001). Mir-1307 and miR-375 together significantly enhanced the diagnostic capacity of HE4, with an AUC of 0.874 (Fig. 4h, *P* < 0.0001). Moreover, we found that combining both miR-1307 and miR-375 with CA125 generated an AUC = 0.977 (Fig. 4g, *P* < 0.0001), meaning that this could serve as a predictive model to distinguish OC from benign ovarian diseases.

**Table 3:** A summary of the diagnostic power of miRNAs, conventional biomarkers, and the combination of the new and conventional markers. AUC = Area under the ROC curve. CI = Confidence Interval

	AUC	95%CI	*P*value	sensitivity%	Specificity%
CA125	0.905	0.834 - 977	<0.0001	79.41%	93.33%
HE4	0.820	0.716 - 0.923	<0.0001	77.50%	86.21%
Mir-1307	0.671	0.550 - 0.791	0.0054	33.33%	94.29%
Mir-375	0.788	0.665 - 0.890	<0.0001	61.76%	87.88%
Mir-1307+Mir-375	0.837	0.725 - 0.917	<0.0001		
HE4+CA125	0.937	0.857 - 0.983	<0.0001		
Mir-1307+CA125	0.959	0.873 - 0.981	<0.0001		
Mir-1307+HE4	0.857	0.730 - 0.919	<0.0001		
Mir-1307+CA125+HE4	0.970	0.892 - 0.997	<0.0001		
Mir-375+CA125	0.940	0.870 - 0.989	<0.0001		
Mir-375+HE4	0.857	0.792 - 0.960	0.0012		
Mir-375+CA125+HE4	0.945	0.904 - 0.999	<0.0001		

### Association of circulating miR-1307 and miR-375 with clinical characteristics in OC patients

To further investigate whether circulating levels of miR-1307 and miR-375 were related to clinicopathological characteristics of OC, we stratified the OC cases by age, tumor stage, localization, and lymph node metastasis. The relationships between miR-1307, miR-375 expression levels, and clinicopathological parameters are summarized in Table 4. Our results suggested that serum exosomal miR-1307 expression was significantly higher in patients with tumor stages (III + IV) than stages (I + II) (*P* = 0.0455). However, there was no significant difference in serum miR-1307 expression among localizations (*P* = 0.9579) or lymph node metastasis (*P* = 0.9000) in OC. As for expression level of serum exosomal miR-375, the differences were not statistically significant for either tumor stage (*P* = 0.5625) or localization (*P* = 0.4725), but the expression level was correlated with lymph node metastasis (*P* = 0.0376). Furthermore, both serum CA125 (*P* = 0.0215) and HE4 (*P* = 0.0494) were related to tumor stages.

**Table 4:** Correlations between Serum exosomal miRNAs markers and clinicopathological characteristics of ovarian cancer

Group	Tumor stage (I+II vs. III+IV)	Localization (Unilateral vs. Bilateral)	Lymph node metastasis (Yes vs. No)
	*P*-value		
miR-1307	0.0455	0.9579	0.9000
miR-375	0.5625	0.4725	0.0376

## Discussion

OC has the fifth highest mortality rate among malignant tumors in women. Screening methods during the early stages are extremely significant for obtaining a good prognosis. CA-125 and HE4 are the most commonly used tumor markers in OC. In our study, the AUC of CA125(0.906 vs.0.893)and HE4(0.82 vs. 0.889) achieved similar result to previous report [33]. Previous research has suggested that females with non-ovarian cancer who have abnormal levels of CA-125 have an increased risk of premature mortality, and that elevated CA-125 can indicate subclinical ovarian disease, such as ovarian cysts, luteal cysts [7, 34].. CA-125 is elevated not only in gynecological disease but also in lung cancer, endocrine and digestive diseases, and nutritional metabolic diseases [35]. HE4 as a traditional tumor biomarker can also indicate abnormalities associated with pathologies besides gynecological disease, for example acute and chronic renal dysfunction [36]. All of the above illustrate the non-specificity of CA-125 and HE4 for OC.

In recent years, numerous studies have confirmed the potential of miRNA in tumor diagnosis and prognosis [37]. Considering the feasibility of tissue sampling and the stability of miRNA, we chose to focus on the exosomal miRNA associated with OC. Using a combination of the clinical relevance and the potential reported in the literature, six miRNAs (miR-1307, miR-214, miR-130a, miR-130b, miR-146, and miR-375) were selected as candidates.

The analysis of the relative expression levels of serum exosomal miRNA in our study revealed that OC cases had significantly higher levels of exosomal miR-1307 and miR-375 compared to individuals with benign tumors or healthy subjects, which is in agreement with the results of previous in vitro studies using OC tissue and cell lines [38, 39]. The expression pattern in urine is still unclear. There are a number of possible reasons why the four other miRNAs yielded insignificant results. The differential expression of exosomal miRNA was not always consistent with the serum miRNA level; this may have been due to selective packaging of exosomes. For example, serum miR-141 and miR-149 were undetectable in serum exosomes from ovarian cancers [40]. In addition, the stability differences of miRNA, the discrepancy of the validation cohort in clinical characteristics, the sample size, and the conditions and storage times of samples also need to be taken into account. The combination of serum biomarkers and serum miRNA is widely employed in clinical research, but uniting serum biomarkers and serum exosomal miRNA remains in the initial stages of development. Previous studies have shown that the diagnostic power of a biomarker panel is generally superior to that of a single marker. Four circulating miRNAs (miR-7, miR-25, miR-93, and miR-429) could provide a significant increase of the AUC (0.98) in OC diagnostics [31]. Other studies have demonstrated the abnormal expression of exosomal miRNAs (miR-23a, miR-92a, miR-21, miR-100, and miR-320) [41]. In our ROC analysis, exosomal miR-1307 and miR-375 demonstrated independent diagnostic power for OC, and the panel consisting of miR-1307 and miR-375 established a significantly greater AUC than for either miRNA individually. The combination of the conventional biomarkers CA125 and HE4 with miR-1307 and miR-375 could yield remarkably increased diagnostic power. All of the evidence together indicates a class of good predictive properties for exosomal miRNA. Furthermore, adding new nucleic markers to traditional biomarkers may provide an effective strategy for non-invasive tumor screening and specific diagnostics**.**

Previous studies have observed the abnormal expression level of miR-1307 in OC [42, 43]. Zhou et al. found there was no statistically significant association between tissue miR-1307 level and tissue differentiation status, clinical stage, or lymph node involvement. However, the clinicopathological analysis in the present study found that miR-1307 was associated with tumor stage. This discrepancy was probably due to the different sample types and sample sizes. Exosomal miRNA has been reported to participate in tumorigenesis, and miR-1307 has been reported to be a regulator for OC chemoresistance via targeting of ING5 in OC cells [39]. Zheng et al. suggested that down-regulation of ING5 might be involved in ovarian carcinogenesis [44]. Further studies confirming that the dysregulation of exosomal miR-1307 can affect OC are necessary. Recently, numerous studies concerning the mechanism of miR-375 in many types of malignancies, including ovarian cancer, have been conducted. Shao et al. suggested that over-expression of miR-375 sensitized the ovarian cancer cells to RAWQ01 by inducing apoptosis. A mechanistic study showed that cellular and exosomal miR-375 could be upregulated by AE, and the altered miR-375 downregulates pro-angiogenic molecules in ovarian cancer cells [45].The results of this study also demonstrated the significant correlation between miR-375 and lymph node metastasis in OC. In addition, the potential of miR-375 as a biomarker has also been reported in osteosarcoma [46] and non-small cell lung cancer [47]. Downregulation of miR-375 was reported to suppress esophageal cancer cell growth and invasion [48], and Zhao et al. found that miR-375 functioned as a tumor suppressor by targeting YWHAZ [49]. These findings together may help to clarify the application value and provide clues for further understanding the mechanism of mir-375 in OC. Together, serum exosomal miR-1307 and miR-375 were shown to have significant potential as targets of OC chemoresistance.

Tumor biomarker are now the focus of tumor research, recent studies have discovered various potentials of tumor biomarker in Gynecological Oncology in addition to the diagnostic. For example, p16 INK4a protein has been reported as the progression/ regression tumour marker in LSIL cervix lesions [50]. Other studies reported the combinations like five-DNA methylation panel [51], the circulating miRNA panel [40], serum microRNA and CA-125panel [52], CPG islands methylation [53] are of great significance in tumor diagnostic efficacy, prognosis, surgical guidance, epigenetic therapy. Tumor biomarkers are involved in molecular signaling pathways that regulate tumorigenesis, metastasis, apoptosis. The aberrant global DNA hypermethylation induced by ERα enhanced anticancer drug resistance in human breast cancer cells by activating the DNMT1 gene [54]. MUC16 encoded protein CA125 moderates TRAIL-induced apoptosis by decreasing TRAIL receptor R2 expression in epithelial ovarian cancer. As a conventional biomarker, CA125 expression were also regulated by miRNAs. Radhakrishnan P et al. reported that the post-transcriptional expression level of MUC16 (CA125) could be regulated by miR-200c in Human Pancreatic Cancer [55]. However, no studies have shown that miR-375, miR-1307 can mediate CA125 expression in OC.

In conclusion, the present study has demonstrated the stable overexpression of serum exosomal miR-1307 and miR-375 and their diagnostic power in OC. A panel of miR-1307 and miR-375 was shown to be effective as a non-invasive diagnostic technique in OC. In addition, exosomal miR-1307 and miR-375 significantly increased diagnostic accuracy for OC compared to conventional biomarkers. Moreover, the mechanisms of exosomal miR-1307 and miR-375 in oncogenesis and chemoresistance are worthy of in-depth study.
